# Evidence of rickettsiae in Danish patients tested for Lyme neuroborreliosis: a retrospective study of archival samples

**DOI:** 10.1186/s12879-018-3210-x

**Published:** 2018-07-11

**Authors:** Lukas Frans Ocias, Ram Benny Dessau, Anne-Mette Lebech, Charlotte Sværke Jørgensen, Randi Føns Petersen, Karen Angeliki Krogfelt

**Affiliations:** 1 Department of Bacteria, Parasites and Fungi, Statens Serum Institut, Artillerivej 5, Copenhagen, Denmark; 2 Department of Clinical Microbiology, Slagelse Hospital, Slagelse, Denmark; 3 Department of Infectious Diseases, Rigshospitalet, Copenhagen, Denmark; 4 Department of Virus and Microbiological Special Diagnostics, Statens Serum Institut, Copenhagen, Denmark

**Keywords:** Rickettsioses, Tick-borne infections, Tick-borne pathogens, Co-infection, Lyme neuroborreliosis, Neuroinfection

## Abstract

**Background:**

With a prevalence of 4.7–13% in Danish *Ixodes ricinus* ticks, *Rickettsia helvetica* is one of the most frequently detected tick-borne organisms in Denmark. Most reports of human exposure have described asymptomatic seroconversion or a mild, self-limiting flu-like illness but it has also been implicated as a cause of subacute lymphocytic meningitis. Because *Borrelia burgdorferi* sensu lato (*Bbsl*) and *R. helvetica* are both found in the same tick species, potential co-transmission is a possibility. We examined 1) the seroprevalence of anti-rickettsia antibodies in patients investigated for Lyme neuroborreliosis (LNB), and 2) the cerebrospinal fluid (CSF) and sera of same patients for the presence of *Rickettsia* DNA.

**Methods:**

Ninety-nine sera and 87 CSF samples from patients with intrathecal synthesis of anti-Borrelia antibodies and 101 sera and 103 CSF samples from patients with no detectable intrathecal synthesis were retrospectively examined for this study. Sera were analyzed for antibodies against spotted fever group (SFG) rickettsiae and both the CSF and sera were tested for *Rickettsia* DNA using a genus-specific real-time PCR.

**Results:**

Of the patients tested for LNB, 32% (64/200) had IgG antibodies against SFG rickettsiae. Among patients with confirmed intrathecal synthesis of Borrelia-specific antibodies, 38% (38/99) exhibited IgG antibodies. None of these values were statistically significant when compared with sera from healthy blood donors (*p* = 0.7 and 0.19). *Rickettsia* DNA was found in the CSF of 4% (8/190) of patients.

**Conclusion:**

No statistically significant difference was found in the seroprevalence of anti-rickettsia antibodies in patients tested for LNB and healthy blood donors, indicative of a low rate of exposure in this group of patients. Eight patients showed evidence of *Rickettsia* DNA in the CSF, five of whom had LNB. However, cycle threshold (Ct) values were high, indicating low concentrations of DNA, and no apparent alteration in the clinical manifestations of LNB were noted in the medical records of these patients.

## Background

With an annual incidence of over 3/100,000, Lyme neuroborreliosis (LNB) is the commonest bacterial neuroinfection in Denmark [1]. It is caused by spirochetes in the *Borrelia burgdorferi* sensu *lato* (*Bbsl*) complex, transmitted to humans by the tick vector *Ixodes ricinus*, also known as the castor bean tick. Apart from *Bbsl*, several other potentially pathogenic organisms have been identified in Danish *I. ricinus* ticks, including *Anaplasma phagocytophilum*, *Babesia* spp., tick borne encephalitis virus (TBEV), *Rickettsia helvetica* and most recently, *Rickettsia monacensis* [2–6].

The *Rickettsia* genus consists of small, aerobic, obligately intracellular Gram-negative organisms and can be divided into two serotypically distinct groups: the spotted fever group (SFG) and the typhus group (TG). Members of the SFG rickettsiae can be found across the globe and are known for causing infections of varying pathogenicity, ranging from the mild African tick-bite fever (*Rickettsia africae*), endemic in sub-Saharan Africa and the West Indies, to the more severe and potentially fatal Rocky Mountain spotted fever (*Rickettsia rickettsii*) in the Americas. Of the two *Rickettsia* spp. existing endemically in Denmark, *R. helvetica* is the most prevalent and is also one of the most frequently encountered tick-borne organisms in the country [3–5]. Most patients exposed to this organism display subclinical infection or a mild, self-limiting febrile illness with headache and myalgia, only rarely accompanied by the often characteristic eschar or rash seen in other forms of SFG rickettsioses [7–10]. However, it has also been implicated as a cause of subacute lymphocytic meningitis [11, 12].

Because *R. helvetica* is transmitted by the same tick vector as the *Bbsl* complex, co-transmission of both agents is a possibility and has been shown to occur in humans [13–15]. Recently, a Dutch study examining the presence of rickettsiae in the cerebrospinal fluid (CSF) of 208 patients suspected of LNB, detected *R. helvetica* and *R. monacensis* DNA in four and one of the patients, respectively [14]. Apart from *R. helvetica*, meningitis has also been associated with several other species of SFG rickettsia including *R. rickettsii*, *Rickettsia conorii*, *Rickettsia japonica* and *Rickettsia felis*, reflecting the neuroinvasive potential in at least some of the species [16–20].

As *Rickettsia* spp. are intrinsically resistant to penicillin, the treatment of choice for Lyme borreliosis, the clinical significance and incidence of co-transmission needs to be further examined in a Danish population. The aim of this study was threefold; 1) to investigate the seroprevalence of antibodies against SFG rickettsia in patients investigated for LNB 2) to examine the presence of *Rickettsia* DNA in the CSF and sera of same patients, and 3) to examine the clinical significance of such a finding.

## Methods

### Study cohort

**Fig. 1:**
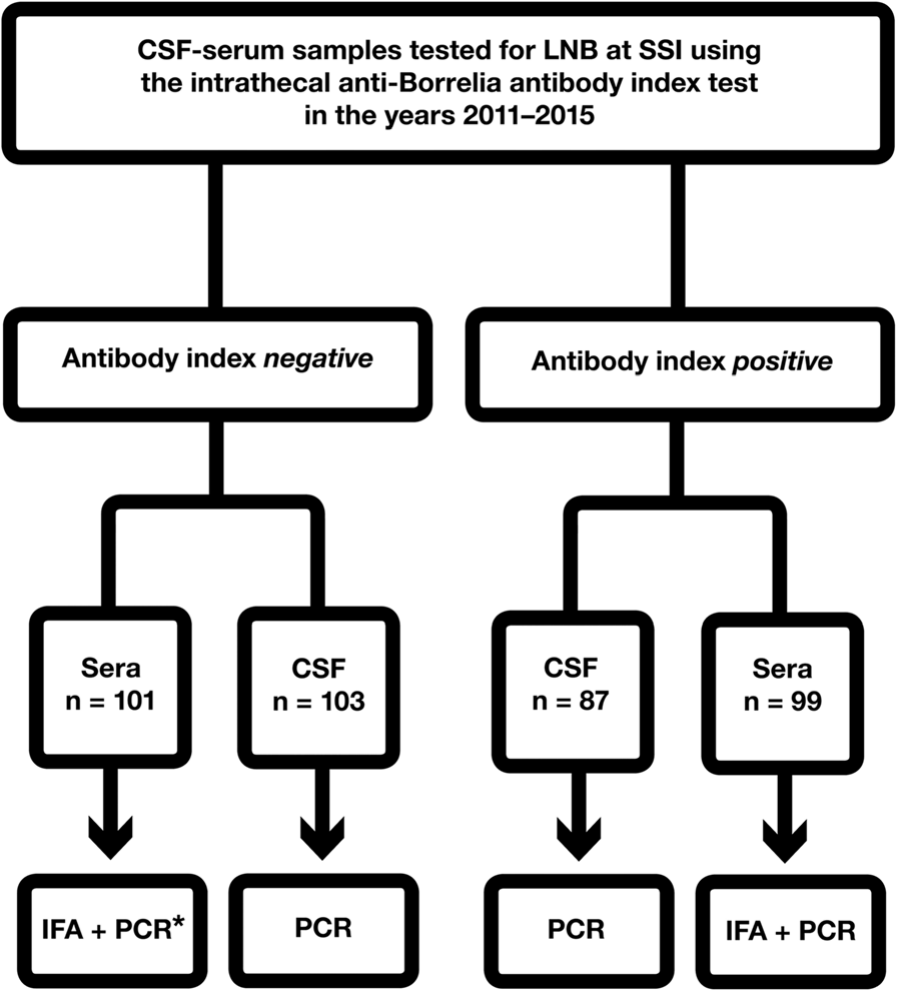
Study flow-chart. CSF; cerebrospinal fluid; PCR; polymerase chain reaction; IFA; indirect immunofluorescense assay. * = Six of the AI negative sera samples contained insufficient volume for PCR analysis and were therefore not analyzed using this method

The study cohort consisted of archival samples of paired sera and CSF from patients tested for LNB using the intrathecal anti-Borrelia (IgM/IgG) antibody index (AI) test. One-hundred and four paired samples were selected from AI positive patients, indicating certain prior exposure to *I. ricinus* ticks, and 110 paired samples were selected from AI negative patients. As some of the archival samples only had small remaining volumes, insufficient for further testing, some of them had to be discarded. A total of 99 sera and 87 CSF samples from AI positive patients, and 101 sera and 103 CSF samples from AI negative patients, remained for inclusion in this study after duplicates and samples containing insufficient volumes had been removed (Fig. 1).

All samples were collected in the months of June to November during the years 2011–2015 and have been stored at the State Serum Institute (SSI) at − 20 °C. The serological analysis for LNB was during these years mostly centralized at SSI and the samples thus represent patients from across the whole country.

### Intrathecal anti-Borrelia antibody index test

The intrathecal anti-Borrelia antibody index test had been performed using an in-house capture enzyme-linked immunosorbent assay (ELISA) that uses native purified flagellum from *B. afzelii* (strain DK1) as antigen [21]. The AI had been calculated using the formula: (ODcsf/ODserum)*(ODcsf − ODserum), with ODcsf and ODserum representing the optical density in CSF and sera, respectively. An AI ≥0.3 is regarded as positive.

### Rickettsia serology

The sera were examined for antibodies against SFG rickettsiae using a commercially available indirect immunofluorescense assay (IFA) (IF0100G, *Rickettsia* IFA IgG; Focus Diagnostics, Inc., Cypress, CA, USA). Inactivated *R. rickettsii* (RR) was used as antigen to detect SFG rickettsiae. The samples were screened at dilutions of 1:64 (IgM) and 1:128 (IgG) and were titrated to end-point fluorescence in two-fold dilutions. A titer of 1:64 or above was considered positive for IgM. The cut-off for IgG had previously, for diagnostic purposes, been raised to 1:512, following testing of healthy Danish blood donors, to achieve a specificity of 95% and reduce cross-reactivity with other bacteria [22]. However, as this elevated cut-off likely results in a loss of sensitivity, a lower cut-off of 1:128 was used in this study. One hundred and eleven serum samples from healthy Danish blood donors served as controls for IgG and 60 blood donor samples served as controls for IgM. These donor samples provided an estimate of the background seroprevalence of anti-rickettsia antibodies in the Danish general population.

### Rickettsia PCR

All of the CSF samples and all, but six, of the sera were examined using real-time PCR for the presence of *Rickettsia* DNA. All samples were centrifuged for 10 min at 15,000 g, after which part of the supernatant was discarded leaving a volume of 200 μl along with the pellet. Extraction of DNA was achieved using the Qiagen QIAamp DNA Mini Kit (Qiagen Danmark, Copenhagen, Denmark), and DNA was eluted into a final volume of 75 μl. All extractions were performed in a hooded bench with laminar air flow and caution was exercised to prevent contamination. A genus-specific real-time PCR with a reported sensitivity of one copy of *Rickettsia* DNA per reaction, was used to amplify a 74 bp fragment of the *R. rickettsii* citrate synthase (CS) gene (gltA) using the primers CS-F and CS-R and the probe CS-P [23]. This assay has previously been used to detect *Rickettsia* spp., including *R. helvetica*, in Danish *I. ricinus* ticks and previous quality assurance programs with the Australian Rickettsial Reference Laboratory have shown perfect matches with no false positive results [5]. Furthermore, the primers used are highly specific and have been validated with no detectable cross-reactivity on medically important bacteria as well as other members of the order *Rickettsiales* including *A. phagocytophilum* and *Bartonella* spp. [23]. One-hundred and three CSF samples from patients examined for other neuroinfections than LNB, primarily encephalitis/meningitis caused by the herpes simplex virus (HSV) or varicella zoster virus (VZV), served as negative controls (Table 1). DNA from *Rickettsia australis* (strain JC) in three 10-fold dilutions served as positive controls.

**Table 1:** Sample characteristics of negative control group (*n* = 103) consisting of CSF, with and without pleocytosis, from patients examined for other neuroinfections than LNB

	Control samples (n = 103)
Median age (IQR, range):	51 (33–63, 0–92)
CSF pleocytosis:	
≤ 5 × 10^6^/L, n:	82
> 5 × 10^6^/L, n (median, IQR, range):	21 (14, 9–65, 6–4248)
CSF protein:	
≤ 0.50 g/L, n:	64
> 0.50 g/L, n (median, IQR, range):	39 (0.69, 0.56–0.99, 0.51–2.30)

*IQR* interquartile range, *CSF* cerebrospinal fluid

### Statistical analysis

Statistical significance was calculated with Fisher’s exact test (two-tailed) and the two-sample Wilcoxon rank sum test using the statistical software R Studio (v1.0.153) [24]. A *p*-value ≤0.05 was considered to be statistically significant.

## Results

### Serology

Antibody titers for the AI positive patients, AI negative patients and healthy blood donors are presented in Table 2. When using a cut-off of 1:128 (one titer above the one suggested by the manufacturer), 64 of the 200 patients (32%) tested for LNB had detectable IgG antibodies and four (2%) had detectable IgM antibodies against SFG rickettsiae. The IgG seroprevalence was 38% (38/99), 26% (26/101) and 30% (33/111) in the AI positive patients, the AI negative patients and the healthy blood donors, respectively. The seroprevalence was not significantly elevated in the AI positive and AI negative patients compared with healthy blood donors (*p* = 0.19 and 0.54). However, a two-sample Wilcoxon rank sum test revealed a non-significant tendency to higher titers in the AI positive group (*p* = 0.08).

**Table 2:** Antibody titers against SFG rickettsiae in the AI positive group (a), the AI negative group (b), and the healthy blood donors (c)

		Antibody index positive (*n* = 99)					
a.	1:64	1:128	1:256	1:512	1:1024	1:2048	1:4096
RR IgM	1	0	0	1	0	0	0
RR IgG	–	12	14	7	5	0	0
b.		Antibody index negative (*n* = 101)					
RR IgM	0	2	0	0	0	0	0
RR IgG	–	9	13	1	1	2	0
c.		Blood donors (*n* = 111/60)					
RR IgM	1	0	0	1	0	0	0
RR IgG	–	18	10	4	1	0	0

When using the routine, diagnostic cut-off of 1:512 [22], 12 patients in the AI positive group and five of the healthy blood donors exhibited anti-rickettsia antibodies. This difference was, however, also non-significant (*p* = 0.07).

### PCR

At the genus-level, *Rickettsia* DNA was identified in eight of 190 (4%) archival CSF samples, with cycle threshold (Ct) values ranging between 39 and 44. Compared with the 103 negative controls, this is a non-significant difference (*p* = 0.054). However, five of these patients were AI positive, reflecting a significantly increased probability of detecting *Rickettsia* DNA in this subgroup compared with the controls (*p* = 0.02). Sequencing was attempted but failed on all eight samples. None of these patients displayed anti-Rickettsia antibodies in the corresponding sera. No CSF in the negative control group, regardless of biochemical findings, showed evidence of *Rickettsia* DNA and no *Rickettsia* DNA could be detected in the sera. All internal negative controls remained negative and all positive controls displayed positive reactions.

### Cases

Hospital medical records were reviewed for the eight patients in whom *Rickettsia* DNA was detected in the CSF. The clinical data are summarized in Table 3. Five of the patients had CSF pleocytosis with mononuclear predominance and typical clinical signs indicative of LNB including peripheral facial palsy and radiculitis, sometimes accompanied by fever or signs of meningismus like photophobia or nuchal rigidity. All five of these patients were treated with intravenous ceftriaxone. Out-patient follow-up 1 month after discharge was performed in three of the patients revealing complete recovery in two of the patients (Case 1 and 2) and slight persisting headache and fatigue in the third (Case 5). One patient had a PCR-verified enteroviral meningitis with fever, headache and neck pain accompanied by an unspecified rash but had recovered completely and was free of symptoms at hospital discharge. Two patients (Case 6 and 7) had no CSF pleocytosis and presented with neurological symptoms.

**Table 3:** Clinical data on patients with detected *Rickettsia* DNA in CSF

	Case 1	Case 2	Case 3	Case 4	Case 5	Case 6	Case 7	Case 8
Co-morbidity	Asthma Esophageal hernia	None	None	Colitis ulcerosa	None	None	Spinal stenosis	None
Symptoms	- Bilateral peripheral facial palsy - Radiculitis	- Unilateral peripheral facial palsy - Headache	- Unilateral peripheral facial palsy - Lower back pain - Radiculitis	- Radiculitis - Erythema migrans - Myalgia	- Radiculitis - Severe headache - Pain in neck and shoulder girdle - Fever - Photophobia	- Transient hemiparesis	- Sensorimotor polyneuropathy	- Fever - Headache - Pain in neck and shoulder girdle - Rash
Clinical diagnosis	LNB	LNB	LNB	LNB	LNB	Transient cerebral attack	–	Enteroviral meningitis (PCR-verified)
SFG *Rickettsia* IFA	NEG	NEG	NEG	NEG	NEG	NEG	NEG	NEG
Intrathecal Borrelia-specific antibody synthesis (index ≥ 0.3)	YES	YES	YES	YES	YES	NO	NO	NO
CRP	5.3	< 1	< 1	6.7	< 1	1	2	**130**
CSF-leukocytes	**34**	**100**	**863**	**192**	**300**	2	1	**133**
CSF-protein	**1.01**	0.42	**1.95**	**0.61**	**1.36**	0.36	**0.56**	**0.52**
CSF-glucose	3.7	3.6	**1.8**	2.9	2.9	3.7	–	3.0
RT-PCR Ct value (CSF)	41	40	39.5	42	39.6	39	43.5	43.5
RT-PCR (sera)	NEG	NEG	NEG	NEG	NEG	NEG	NEG	NEG
Antibiotic treatment	IV ceftriaxone + IV acyclovir	IV ceftriaxone	IV ceftriaxone	IV ceftriaxone	IV ceftriaxone	–	–	IV ceftriaxone + IV acyclovir
Follow-up	Asymptomatic 1 month post treatment	Asymptomatic 1 month post treatment	Clinical follow-up data missing.	Well-being at hospital discharge.	Responds to treatment. Slight headache and fatigue 1 month post hospital discharge.	Remits after 36 h with full recovery of neurological function.	Symptoms persistent for several years.	Asymptomatic at hospital discharge

Abnormal values are printed in boldface. All patients with CSF pleocytosis had mostly mononuclear leukocytes. The cut-offs used for SFG *Rickettsia* IFA were 1:64 for IgM and 1:128 for IgG. Reference values: CRP: < 10 mg/L. CSF-leukocytes: < 5 × 10^6^/L. CSF-protein: 0.15–0.50 g/L. CSF-glucose: 2.2–3.9 mmol/L

*SFG* spotted fever group, *IFA* indirect immunofluorescense assay, *LNB* Lyme neuroborreliosis, *CSF* cerebrospinal fluid, *PCR* polymerase chain reaction

One of the patients (Case 5) had, in the months prior to hospital admission, experienced a tick bite in Sweden. No other patients had any mention of international travel, leading up to the hospital admission. As *R. helvetica* is also the most common tick-borne rickettsia found in Sweden, all patients had thus most likely been infected with this species or possibly, *R. monacensis* [25].

## Discussion

We found no significant difference in the seroprevalence of antibodies against SFG rickettsiae between patients investigated for LNB and healthy blood donors and no *Rickettsia* DNA in the tested sera, indicating low rates of exposure to SFG rickettsiae in Danish patients with Lyme borreliosis. Eight patients showed evidence of *Rickettsia* DNA in the CSF, five of whom were Borrelia AI positive, reflecting a significantly increased probability of detecting *Rickettsia* DNA in the AI positive subgroup compared with patients tested for other neuroinfections than LNB (*p* = 0.02). Despite this, the AI positive patients presented with typical clinical signs of LNB with no apparent alteration in the clinical manifestations of this infection.

### Serology

The high seroprevalence of IgG antibodies against SFG rickettsiae among healthy blood donors is indicative of considerable non-specific background reactivity, revealed by the lowering of our titer cut-off for this study. Nevertheless, the absence of a significant difference in the seroprevalence of antibodies between healthy blood donors and patients examined for LNB, regardless of titer cut-off and Borrelia AI, is indicative of low rates of exposure in this group of patients.

Important caveats to be emphasized are that the precise time elapsed from being bitten to being tested is not known and that the precise kinetics of the human antibody response to *R. helvetica* and *R. monacensis* have not been studied, factors that could potentially impact the current results. However, considering that adult Danish patients with LNB are often diagnosed late in the course of their disease with a median delay of 20 days from the start of neurological symptoms until first hospital contact, one can assume that enough time had passed to allow seroconversion in most of the AI positive patients exposed to SFG rickettsiae [26].

Lastly, *R. rickettsii*, and not *R. helvetica*, was used as IFA antigen in the current study. *Rickettsia conorii* is often used as antigen to screen for SFG rickettsiae and cross-reactivity between this species and *R. helvetica* has previously been demonstrated [7, 14]. As *R. rickettsii* is phylogenetically closely related to *R. conorii* and due to the generally considerable homology between SFG rickettsiae resulting in high levels of serological intra-group cross-reactivity, we expect *R. helvetica* to be detectable using this current assay, as has been suggested by a previous study [27]. This has been corroborated by preliminary tests on Norwegian sera from patients seropositive for *R. helvetica*, indicating that these antibodies are detectable using *R. rickettsii* as antigen and the current cut-offs (unpublished data).

### PCR

Whether the detected *Rickettsia* DNA represents true neuroinfection or not can be discussed. All internal negative controls remained negative, making contamination less likely. Furthermore, *Rickettsia* DNA was not detected in the negative control group, consisting of patients tested for other neuroinfections than LNB and representing a range of varying biochemical values, reducing the chances of it being an artifact. Sequencing was attempted but failed on all eight samples, most likely due to the small amounts of bacterial DNA in the samples, as reflected in the high Ct values. However, the general lack of discerning clinical features in these cases and the apparent lack of pleocytosis and clinical signs of infection in two of the subjects necessitates cautious interpretation of these PCR findings. It is important to note that none of the patients received treatment with doxycycline (Table 3). Despite *Rickettsia* spp. being inherently resistant to beta-lactam antibiotics [28] and these patients thus never receiving any effective treatment against their putative *Rickettsia* infection, all patients with spinal fluid pleocytosis and available follow-up data recovered from the acute phase of their infections, with two of them experiencing a full recovery 1 month post treatment. One of the previously described cases of putative *R. helvetica* neuroinfection had concomitant PCR-verified herpes simplex meningitis and it is, likewise, unknown what role *R. helvetica* played in this infection and how the patient would have fared had he not received treatment with doxycycline [12]. Furthermore, it is important to emphasize that detection of bacterial DNA is not equivalent to detecting the organism itself, and the present study does not establish any causal relationship between these findings and the clinical features of the patients.

Interestingly, none of the patients exhibiting DNA in their CSF had detectable antibodies against SFG rickettsiae. Possible explanations could be that the patients were tested prior to the development of anti-rickettsia antibodies, a lack of sensitivity in the IFA assay or sample contamination with rickettsial DNA. It is possible that the lack of antibodies facilitated the detection of *Rickettsia* DNA by allowing the bacteria to survive and spread, as suggested by a recent study [29].

### Strengths and limitations

The study is limited by its retrospective nature and its use of frozen archival specimens of variable volume, ill-suited for culture and lacking convalescent samples for serology. However, the use of archival material also allowed the inclusion of a greater number of patients with a confirmed anti-Borrelia AI, ascertaining prior tick exposure, than would have been possible in a reasonable time frame following a prospective study design. It is important to remember that all samples had initially been sent to a serological laboratory and have thus, not been handled in a clean working environment appropriate for PCR analysis, why potential sample contamination cannot be ruled out. This also applies to the negative CSF controls, many of which had previously been tested for HSV/VZV using an intrathecal antibody index test. However, contamination seems unlikely as we have never amplified or cultured rickettsiae in our serological laboratory and have only used commercial IFA kits with preformed slides, on which the antigen is already fixed, for serological analysis.

The effect of prolonged storage in − 20 °C freezers and multiple cycles of thawing-freezing on the quality and yield of potential bacterial DNA can also be discussed. The former was not considered a major problem as *R. helvetica* has previously been detected in CSF (using both culture and PCR) after having been stored for over a year in a − 20 °C freezers [11]. Regarding the latter, prior studies on *Treponema pallidum* have shown only negligible effects of multiple freezing-thawing cycles on the quality and yield of spirochetal DNA in CSF, and this is likely the case for other forms of bacterial DNA as well [30].

## Conclusion

Despite them being one of the commonest tick-borne organisms in Denmark, antibody reactivity against *Rickettsia* spp. was not significantly increased in patients investigated for LNB as compared with healthy blood donors. The same was true when looking at the subgroup of patients with a positive anti-Borrelia AI test, confirming a prior tick bite. These findings indicate low levels of SFG rickettsiae co-transmission in this group of patients.

Furthermore, eight patients displayed small amounts of *Rickettsia* DNA in the CSF. However, the lack of CSF pleocytosis in two of the patients, the complete or near-complete clinical recovery in most of the patients, despite being withheld doxycycline, and a general lack of clear, discerning clinical features in all of the patients, necessitates cautious interpretation of these results. Should this CSF data indicate true rickettsial infection, such occurrences appear to be rare and of uncertain significance to the patient.

## Abbreviations

AI: Antibody index
*Bbsl*: *Borrelia burgdorferi* sensu lato
CRP: C-reactive protein
CS: Citrate synthase
CSF: Cerebrospinal fluid
Ct: Cycle threshold
DNA: Deoxyribonucleic acid
ELISA: Enzyme-linked immunosorbent assay
HSV: Herpes simplex virus
IFA: Indirect immunofluorescense assay
IQR: Interquartile range
LNB: Lyme neuroborreliosis
LP: lumbar puncture
PCR: Polymerase chain reaction
RR: *Rickettsia ricketsii*
SFG: Spotted fever group
SSI: State Serum Institute
TBEV: Tick-borne encephalitis virus
TG: Typhus group
VZV: Varicella zoster virus
